# Infectious Diseases on the Western Coast of South America

**Published:** 1878-08

**Authors:** Henry M. Lyman


*Editors Chicago Medical Journal and Examiner:*

Ten or fifteen years ago, the journal-reading portion of the medical fraternity were aware of the existence of a live medical periodical, edited in Leavenworth City, by Dr. C. A Logan, a gentleman well known as one of the most eminent physicians west of the Mississippi. Connected with the geological survey of the new State of Kansas, his writings gave evidence of a mind trained by close observation of nature in her widest relations. It was a matter for regret when he ceased to use his pen, and disappeared beyond the horizon of our view. This present writer had long given him up for dead, when at length he appeared once more among the haunts of men—this time, upon the streets of Chicago—after a period not of suspended animation, but of honorable exile as minister of the United States resident in Chili. And now comes this modest little volume, in which our distinguished friend has embodied the ripe results of his observation and thought while dwelling in that far distant and little known land.

Finding himself in a country remarkable alike for its relics of ancient society, and for the physical conformation of its territory, the observing stranger was not long in discovering that even the forms of disease which are there prevalent exhibit the modifying influences of the peculiar conditions under which they occur. Certain diseases, notably the infectious diseases, are almost unheard of in Chili, while those that do occur, like typhoid fever and small-pox, present decisive symptoms of divergence from the European and North American type. Certain diseases seem to have originated spontaneously in that country, while others which are epidemic in Europe and in North America, are either unknown or seem to lose in great measure their infectious properties when introduced among the people who inhabit the Pacific slopes of the Andes. An inquiring mind could not fail to busy itself with a search for the causes of such departures from the types of disease which prevail in other countries; and, accordingly, the doctor has in his little volume set forth his views regarding infectious diseases and their limited prevalence among the inhabitants of the western coast of South America. One of the principal causes of this peculiar immunity is by our author thought to be ozone. This substance is generated in great quantity by the electrical activity, to which he also refers the fearful earthquakes which agitate that portion of the continent. This hypothesis leads the doctor into a series of excursions, in which he sets forth pretty fully his belief concerning these appalling phenomena. We are treated to a chapter on force and energy, in which, though if this were a text book on physics one might regret the absence of the luminous definitions of a Balfour Stewart, the author's meaning is clear enough, and is in accord with the most advanced scientific thought of the day. This assertion may seem strange to the tyros in science, to the men of one text book, but no one can read far or think much without finding himself in the presence of just such problems as Dr. Logan has here brought up before the mind of the reader. The same thing is true of his chapters on the causation of earthquakes. This hypothesis will at once array against him all those who have only a slight acquaintance with the subject, and all those who are partisan exponents of rival hypotheses; but when one examines the literature of the subject, it appears that if one cannot fully agree with the doctor, he is certainly in very good company, and his observations are worthy of very respectful attention. The idea that certain earthquakes are the result of "subterranean lightning," has probably occurred to many observers who have watched the behavior of submarine cables before and after an earthquake shock—behavior which shows beyond question that tremendous electrical disturbance is a concomitant, if not the actual cause of the agitation of the soil. These observations are as yet in their infancy, and it is to be desired that our author may yet conclude to publish his own notes in full, for it is only by careful accumulation and comparison of facts that we can hope to arrive at any degree of certainty regarding this much debated subject.

Having thus cleared the way, our author asserts that in those parts of South America "where the *earthquake energy* is most strongly and constantly developed, the existence of large number of infectious diseases which devastate other parts of the world is unknown; and * * that in such portions * * alone as possess a rainfall during a limited part of the year, are any of these diseases to be observed; and then, perhaps, as a rule, after the winter earthquake has passed." He then disposes of the objection that the isolation of that quarter of the globe may act as a protective quarantine, by showing that direct communication with other countries is now so speedy and constant that isolation no longer exists. The only facts capable of influencing the case, then, are atmospheric aridity, and a highly electrical condition of the earth and air. This at once suggests the idea, that, if bacteria and other similar aerial organisms are the causes of infectious diseases, perhaps the dryness of the air is sufficient to prevent their existence, and so to prevent the occurrence of those epidemic diseases which have been supposed to be caused by their development. But investigation has convinced our author that these microscopical organisms are as abundant in Chili as in other parts of the world; consequently, he draws a conclusion unfavorable to the bacterian theory of infection. The remaining portion of this division of the book is devoted to a brief but interesting sketch of the sanitary peculiarities and prevalent diseases of the Pacific coast of America, a sketch which makes one regret that the author had not devoted a larger space to this part of his subject.

The second half of the volume is occupied with what may correctly be called the Physics of Infectious Diseases. The author here boldly attacks the problem of the origin of such diseases, and the method of their development and propagation. It is here that he is at his best. It is upon this field that he has earned his right to a place among the foremost medical philosophers of our day. This proposition I am ready to maintain, in spite of certain errors of detail into which he may have been betrayed. For example, when one considers the existence of lowly animal forms without a nervous system, the assertion of our author, that "animal life begins in the central nervous axis," seems too exclusive. After a certain stage of evolution has been attained, there is a sense in which this proposition is true; and in its application to the human animal the doctor has on his side such thinkers as President Porter, and some of the ablest men who ever lived. This whole chapter on the Ideal Function of the Nervous System, will undoubtedly arouse a great deal of dissent, much of which, however, will be found to grow out of the difficulty of following so abstruse a discussion when presented in so condensed a form. In like manner, the author, in his chapter on the source of Animal Heat, allies himself distinctly with those philosophers who recognize the existence of a *vital force* which is different from, yet capable of strict correlation with the ordinary physical and chemical forces. To such condensation of expression, however, has he restricted himself in this chapter that probably nine-tenths of his readers will fail to appreciate its real significance. It is this magnificent endeavor after compactness of form and terseness of utterance which forms one of the greatest obstacles to the success of this book as a literary adventure. This, however, is a fault which it shares with the very best of company, so the author must not be discouraged if his work cannot vie in popularity with the latest periodical literature. In fact, it often seems as if those minds which are best adapted to attract popular attention to their work are the least fitted to grapple with the great problems of nature, and are the most likely to rest contented with the shallowest theories of the universe. It is, therefore, positively refreshing to meet with one who has the courage, and the knowledge and the insight necessary to enable him to rank himself with the minority in opposition to the fashionable heresies of the day. This our author does, placing "himself in direct antagonism to those ultra chemists who maintain that there is nothing peculiar in organic chemistry, and that its actions are subject to the same laws as those governing inorganic matter; and who attempt the proof of the correctness of this view by their ability to construct some compounds belonging to the animal body outside of the operation of its peculiar laws." The important results of the chemist "in this direction only prove the existence of a force presiding over the methods of vital chemistry which renders him impotent to construct such eminently vital products as urea; not to speak of the characteristic structures of a healthy body, as muscle, flesh, blood, nerve." Here the doctor may be thought to have laid himself open to attack, for the whole army of small chemists is unwearied in its self-glorification over the artificial production of a body isomeric and isomorphic with urea. The difference between the vital chemistry of the animal cell, and the synthetic chemistry of the laboratory becomes apparent when these two substances are subjected to the action of polarized light, and are found to behave differently in its presence—thereby justifying Dr. Logan in his position.

The same logical subtlety and penetration is evident when the doctor enters upon the discussion of the various theories of fermentation which have been put forth. As might be expected, he sides with Liebig against that arrant sciolist Pasteur; and, after reviewing the hypotheses of bacterial infection, and contagious bioplasts, he brings the reader through a course of masterly reasoning to the conclusion from which there is no escape, that the cause of each infection resides in a specialized molecule, born of abnormal molecular movements in the living tissues of the body, and capable of impressing its unhealthy modes of molecular motion upon other molecules with which it may be brought in contact. This he calls "the infectious molecule." This is the portable agent which may find its way from one body to another, sometimes needing to ripen in the soil of the earth or under water on its way; never reproducing itself like a living "germ," but always potent to disturb every adjacent molecule of protoplasm, and often able to impart that peculiar energy to such adjacent molecules until every particle of animal body may have become thus endowed with the modes of motion and properties characteristic of the original infectious molecule. This is a great conclusion. It is the only one logically possible in the present state of science, and yet it is by no means the fashionable theory of the day.

One of the greatest hindrances to the establishment of sound medical doctrine in these times consists in the fact that so few of the leaders of medical opinion are qualified to form an independent opinion upon a subject like this. Consequently, the majority of physicians are at the mercy of the least little upstart microscopist, who may happen to infest a popular German University. Witness the lamentable delusions now current regarding diphtheria and similar diseases. Witness the crowds of people who go astray in the wake of Pasteur and Lister, and Tyndall and other "blind leaders of the blind." This little book, however, gives encouraging evidence that all have not yet "bowed to the knee of Baal," but that there still remain a chosen few who will yet bring back the medical host to its rightful allegiance. This must necessarily be a tedious undertaking, for, unfortunately, the men who most need the corrective influence of this pregnant little volume, are precisely the very men who will never even make the effort to read beyond its title page.

HENRY M. LYMAN.

